# The cytoplasmic tail of IBV spike mediates intracellular retention via interaction with COPI-coated vesicles in retrograde trafficking

**DOI:** 10.1128/jvi.02164-24

**Published:** 2025-01-22

**Authors:** Rong Liang, Jiaxin Tian, Kangchengyin Liu, Liman Ma, Ruihua Yang, Lu Sun, Jing Zhao, Ye Zhao, Guozhong Zhang

**Affiliations:** 1 National Key Laboratory of Veterinary Public Health Security, College of Veterinary Medicine, China Agricultural University 630101 , Beijing, China; 2 Key Laboratory of Animal Epidemiology of the Ministry of Agriculture, College of Veterinary Medicine, China Agricultural University 630101 , Beijing, China; University of Kentucky College of Medicine, Lexington, Kentucky, USA

**Keywords:** infectious bronchitis virus, spike, cytoplasmic tail, COPI, retrograde trafficking

## Abstract

Coronaviruses are characterized by their progeny assembly and budding in the endoplasmic reticulum-Golgi intermediate compartment (ERGIC). Our previous studies demonstrated that truncation of 9 amino acids in the cytoplasmic tail (CT) of the infectious bronchitis virus (IBV) spike (S) protein impairs its localization to the ERGIC, resulting in increased expression at the plasma membrane. However, the precise mechanism underlying this phenomenon remained elusive. In this study, we provide evidence that the IBV S protein could utilize coatomer protein-I (COPI)-coated vesicles for retrograde transport from the Golgi to the endoplasmic reticulum (ER). We identified the KKSV motif as the critical binding site within the CT domain of IBV S protein for COPI interaction. Further analysis reveals that IBV infection does not modulate host COPI expression. However, when COPI expression is disrupted, a higher proportion of S protein escapes to the plasma membrane. Moreover, inhibition of COPI-mediated transport during viral infection severely impairs progeny virion production and leads to increased S protein accumulation at the plasma membrane, inducing cell-cell fusion and syncytia formation. Our findings contribute to a deeper understanding of S protein intracellular trafficking during coronavirus infection, and offer valuable insights into the molecular mechanisms of viral replication and host cell biology.

**IMPORTANCE:**

Viruses hijack or modify host cellular machinery and associated pathways to facilitate their own replication. Here, we demonstrate that the infectious bronchitis virus (IBV) S protein directly interacts with coatomer protein-I (COPI)-coated vesicles through the KKSV motif in its cytoplasmic tail. COPI-coated vesicles mediate the retrograde transport of S protein from the Golgi apparatus to the endoplasmic reticulum-Golgi intermediate compartment, where viral particle assembly occurs. Our findings not only advance our understanding of IBV S protein trafficking mechanisms but also provide valuable insights for developing more effective vaccine strategies.

## INTRODUCTION

Coronaviruses (CoVs) are enveloped, positive-sense, single-stranded RNA viruses with an extensive host range, capable of infecting various mammalian and avian species (1). Based on serological and genomic characteristics, CoVs are classified into four genera: α, β, γ, and δ. Among these, the γ coronavirus genus includes infectious bronchitis virus (IBV), which causes acute respiratory and urogenital infections in chickens. IBV is globally prevalent, posing significant threats to poultry industries and causing substantial economic losses (2, 3). CoVs primarily encode four structural proteins: the spike (S) protein, which mediates entry into host cells; the nucleocapsid (N) protein, which encapsulates the viral RNA; and the membrane (M) protein and the envelope (E) protein, both of which are involved in viral assembly and budding. The S protein is anchored in the virus envelope and determines the specificity of virus-host cell interactions, viral tropism, and infectivity. It comprises three domains: an extracellular domain, a transmembrane domain, and a short cytoplasmic tail (CT) (4, 5). In infected cells, newly synthesized S protein is initially transported from the ER to the Golgi apparatus. The majority of S proteins are subsequently transported from the Golgi to the endoplasmic reticulum-Golgi intermediate compartment (ERGIC), where they participate in viral assembly and budding (6–8). A portion of S proteins are also transported to the cell surface. Various intracellular transport signals, such as the endoplasmic reticulum (ER) retention signal (ERRS) in the CT of the S protein, have been identified to ensure its retrograde transport from the Golgi to the ERGIC. This signal, often in the form of a di-lysine motif (KKxx, KxKxx, or KxHxx, where x represents any amino acid [aa], KKSV for the IBV), facilitates the binding of S protein to host transport proteins, mediating its retrograde transport to the ERGIC for efficient viral assembly (9–11).

In host cells, membrane trafficking between the ER and the Golgi apparatus is mediated by coatomer protein-I (COPI) and coatomer protein-II (COPII) (12, 13). Anterograde transport of transmembrane and soluble proteins from the ER to the Golgi is driven by COPII, whereas retrograde transport from the Golgi back to the ER is facilitated by COPI (14). COPI is a heptameric complex composed of seven subunits: α, β, β′, γ, δ, ε, and ζ (15). The CT of escaped ER-resident protein cargoes and soluble ER proteins often contain di-lysine motifs that are specifically recognized by COPI subunits. For instance, the KKxx motif is specifically recognized by the α-COPI subunit, while the KxKxx motif is recognized by the β′-COPI subunit (16–18).

Viruses, lacking the organelles present in living cells, often hijack or modify intracellular membrane structures and associated cellular pathways to support their life cycles. Recent studies have revealed that COPI is involved in the replication processes of various viruses (19, 20). Notably, during the COVID-19 pandemic, research demonstrated that the S protein of SARS-CoV and SARS-CoV-2 interacts with COPI via its KxHxx motif, playing a crucial role in S protein trafficking and syncytium formation (9–11, 21). However, it remains unclear whether the KKSV motif in the CT of the IBV S protein can similarly interact with COPI. Moreover, most of the previous studies have primarily been conducted at the plasmid level, leaving the role of COPI in coronavirus infection largely unexplored. Our earlier research indicated that during serial passage of IBV in chicken embryos, premature stop codon mutations frequently occur at the 3′ end of the S gene. These mutations result in the truncation of 9 aa in the CT of the S protein, eliminating the KKSV motif. This truncation impairs the proper localization of the S protein to the ERGIC, causing its mislocalization to the plasma membrane (22). Considering the important role of COPI in the retrograde transport of S protein in other CoVs, we wonder if this phenomenon is related to the change of IBV spike-COPI interaction. In this study, we demonstrate that the IBV S protein directly interacts with COPI-coated vesicles via the KKSV motif in its CT. Under conditions of viral infection, inhibition of COPI-mediated transport results in an increased mislocalization of the S protein to the plasma membrane, thereby impairing the production of progeny virions. This finding provides significant insights into the intracellular trafficking of the S protein during coronavirus infection, enhancing our understanding of viral replication mechanisms.

## RESULTS

### Interaction of IBV spike protein with COPI-coated vesicles

**Fig 1:**
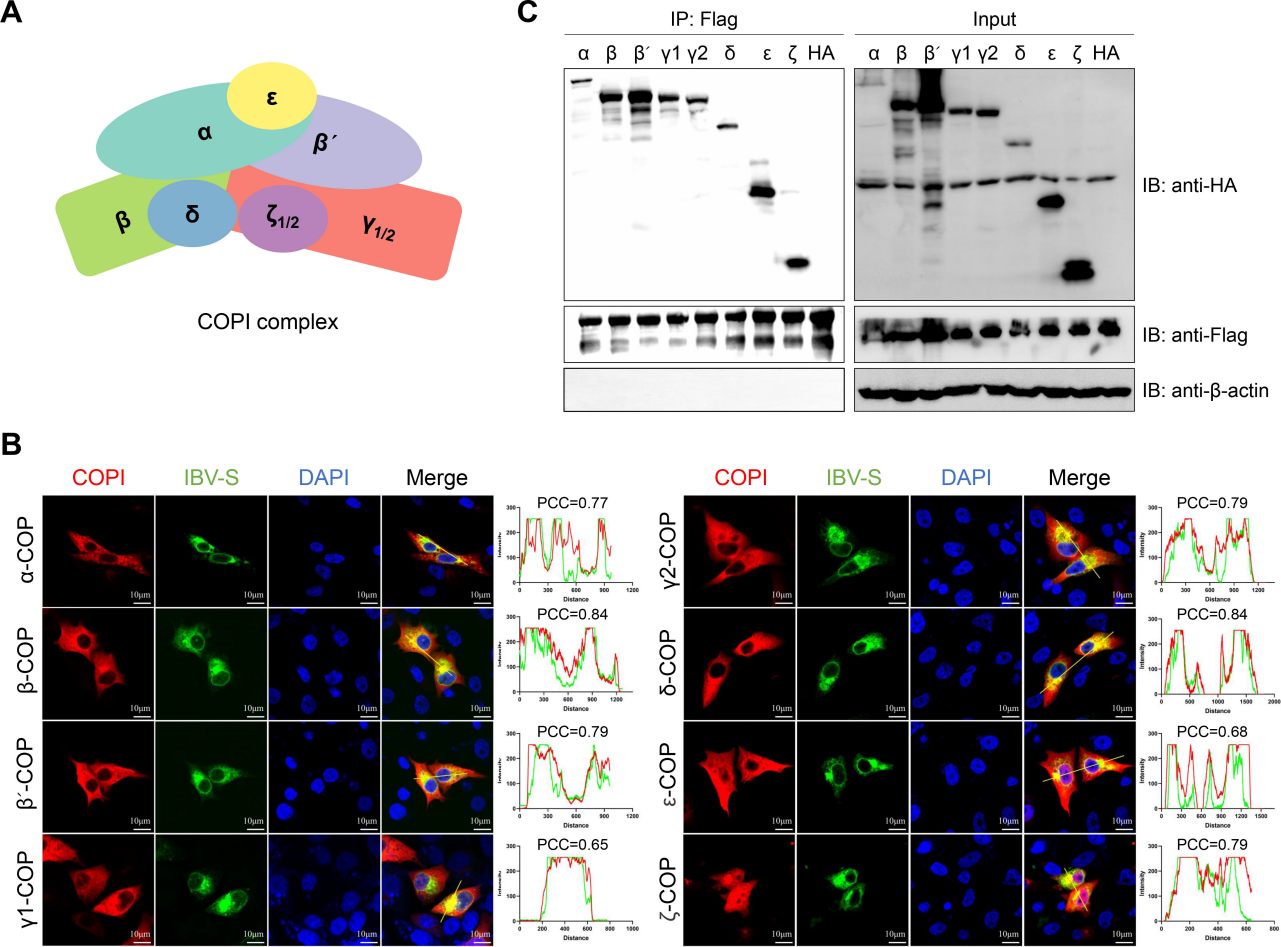
Interaction between the IBV S protein and COPI-coated vesicles. (**A**) Schematic representation of COPI-coated vesicles. (**B**) Immunofluorescence analysis of the interaction between S protein and individual COPI subunits. BHK-21 cells were co-transfected with Flag-tagged S and HA-tagged COPI subunits (α, β, β′, γ1/2, δ, ε, and ζ). At 24 h post-transfection, cells were fixed and immunolabeled with anti-Flag and anti-HA antibodies, followed by incubation with Alexa Fluor 488- and 555-conjugated secondary antibodies, respectively. Nuclei were stained with DAPI. The extent of colocalization of the S protein with COPI subunits was quantified using PCC. Scale bars: 10 µm. (**C**) Coimmunoprecipitation analysis of the interaction between S protein and individual COPI subunits. HEK 293T cells were co-transfected with Flag-tagged S and HA-tagged COPI subunits (α, β, β′, γ1/2, δ, ε, and ζ). At 48 h post-transfection, cell lysates were subjected to immunoprecipitation using anti-Flag M2 affinity gel.

COPI consists of seven subunits, α, β, β′, γ, δ, ε, and ζ, and mediates the retrograde transport of cargos from the Golgi apparatus back to the ER (Fig. 1A). This transport includes host proteins and lipids that have escaped from the ER, as well as certain viral proteins during infection. To determine whether COPI is involved in the transport of the IBV S protein, we constructed eukaryotic expression plasmids for each of the seven COPI subunits. Following transient transfection, immunofluorescence assay (IFA) and coimmunoprecipitation (Co-IP) were used to assess the interaction between the IBV S protein and each subunit. The IFA results demonstrated both S protein and COPI subunits were predominantly positioned in cellular cytoplasm, and a colocalization with each of the COPI subunits (Fig. 1B). To ensure that colocalization was relevant, we next performed Co-IP experiments. In HEK 293T cells, each COPI subunit was co-expressed with the wild-type S protein (S-WT). After 48 h, cell lysates were immunoprecipitated using M2 affinity gel. Consistent with the IFA results, the IBV S protein interacted with each COPI subunit (Fig. 1C). These findings indicate that the IBV S protein can interact with COPI-coated vesicles within the cell.

### S protein interacts with COPA WD-repeat domain via its cytoplasmic tail

**Fig 2:**
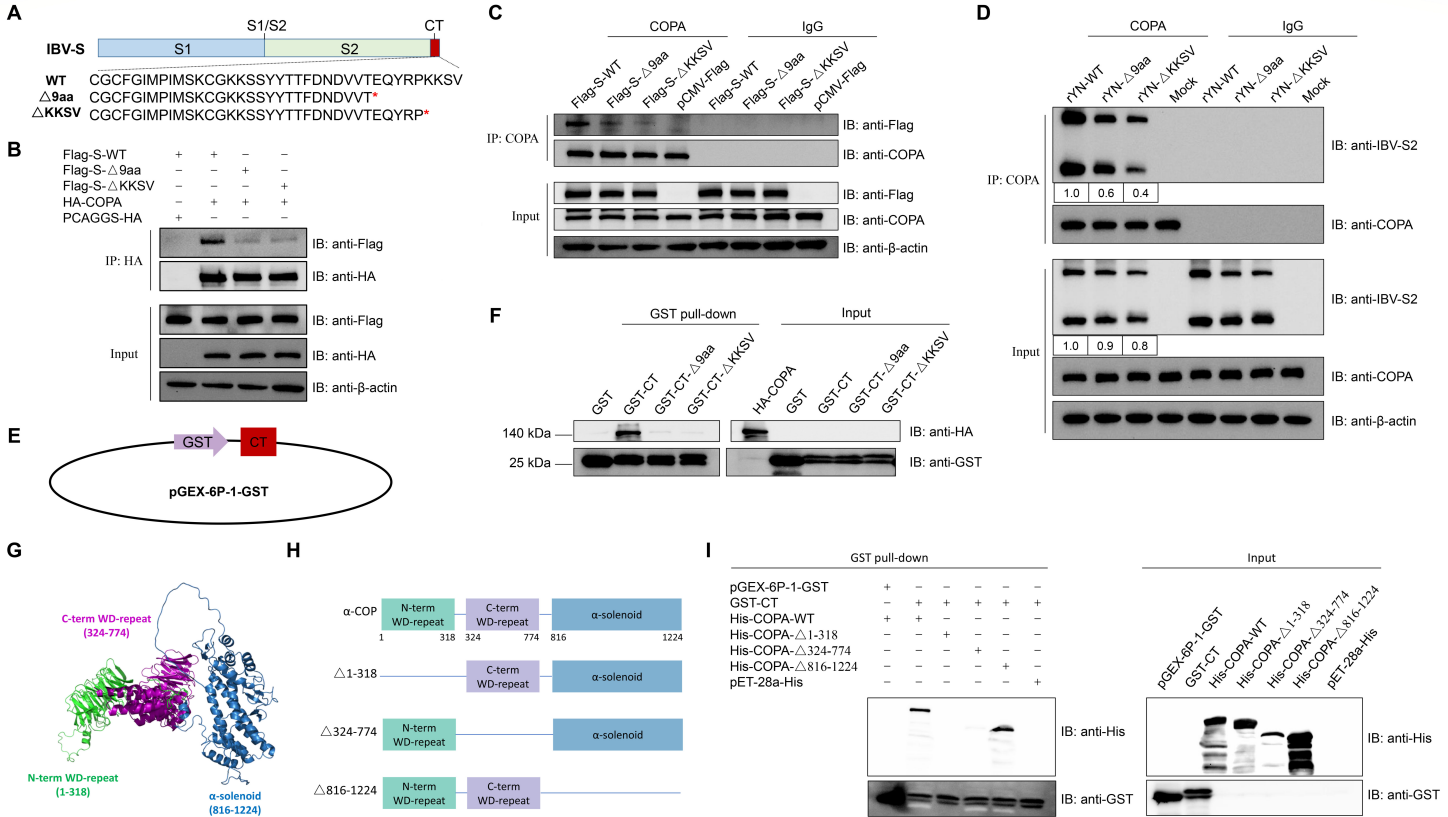
Interaction between the cytoplasmic tail of the IBV S protein and COPA. (**A**) Diagrammatic representation of mutations in the S protein sequences. Mutated residues are indicated in red, with asterisks (*) denoting termination mutations. (**B**) Co-IP analysis of the S protein CT and COPA interaction. HEK 293T cells were co-transfected with Flag-tagged S (S-WT, S-Δ9aa, and S-ΔKKSV) and HA-tagged COPA or empty vector pCAGGS-HA. At 48 h post-transfection, cell lysates were immunoprecipitated using HA-conjugated protein A/G magnetic beads. (**C**) Endogenous Co-IP analysis of the S protein CT and COPA interaction. HEK 293T cells were transfected with Flag-tagged S (S-WT, S-Δ9aa, and S-ΔKKSV). At 48 h post-transfection, cell lysates were immunoprecipitated using COPA-conjugated or IgG-conjugated protein A/G magnetic beads. (**D**) Endogenous Co-IP analysis of the S protein CT and COPA interaction under infection conditions. CEK cells were infected with recombinant IBVs (rYN-WT, rYN-Δ9aa, and rYN-ΔKKSV) at an MOI of 0.01. At 48 h post-infection, cell lysates were immunoprecipitated using COPA-conjugated or IgG-conjugated protein A/G magnetic beads. (**E**) Schematic representation of the GST-CT plasmid construct. (**F**) GST pull-down assays demonstrating direct binding of COPA to the S protein CT. GST or GST-tagged constructs (GST-CT, GST-CT-Δ9aa, and GST-CT-ΔKKSV) were immobilized on glutathione Sepharose 4B and incubated with lysates from cells transfected with HA-tagged COPA. (**G**) Schematic diagram of the COPA domain structure. (**H**) Schematic representation of truncated COPA domain constructs. (**I**) GST pull-down assays demonstrating direct binding of the S protein cytoplasmic tail to the WD domain of COPA. GST or GST-CT were immobilized on glutathione Sepharose 4B and incubated with bacterial lysates containing His, His-COPA-WT, His-COPA-Δ1–318, His-COPA-Δ324–774, or His-COPA-Δ816–1224.

COPI typically recognizes cargo through di-lysine motifs such as KKxx or KxKxx. Our previous research indicated that the deletion of 9 aa or the KKSV motif from the CT of the S protein significantly increased its expression on the cell surface. To investigate whether this phenomenon is associated with altered COPI binding affinity, we explored the interaction between COPI and S protein CT mutants (Fig. 2A). Previous studies have shown that the α subunit of COPI (COPA) primarily recognizes the KKxx motif (23). Therefore, we focused on COPA for subsequent experiments. HEK 293T cells were co-transfected with Flag-tagged S protein (S-WT or mutants S-Δ9aa, S-ΔKKSV) and HA-tagged COPA. As illustrated in Fig. 2B, COPA exhibited the strongest binding affinity for S-WT. While deletion of either the last 9 aa or the KKSV motif from the S protein’s CT significantly attenuated its interaction with exogenous COPA, the empty vector PCAGGS-HA failed to pull down the S protein. Endogenous Co-IP experiments corroborated these findings, showing that CT mutations disrupted the interaction with endogenous COPA (Fig. 2C). Further assessment of the interaction between COPA and S protein mutants under infection conditions showed that the mutants (Δ9aa, ΔKKSV) bound less COPA compared to the S-WT (Fig. 2D). These results suggest that deletion of 9 aa or the KKSV motif weakens the interaction between the S protein and COPA.

To determine whether COPA directly binds to the CT of the S protein, we expressed the 38 aa of the S protein CT in the prokaryotic expression vector pGEX-6P-1 (Fig. 2E). GST pull-down assays demonstrated that COPA directly binds to the 38 aa of the S protein CT, and this binding is disrupted by the deletion of 9 aa or the KKSV motif (Fig. 2F). This indicates that the KKSV motif is crucial for the interaction with COPA.

COPA consists of three domains: N-terminal WD-repeat, C-terminal WD-repeat, and α-solenoid (Fig. 2G). We generated deletion constructs for each domain (Δ1–318, Δ324–774, and Δ816–1,224) and expressed in the pET-28a vector with a His tag (Fig. 2H). GST pull-down assays showed that the CT of the S protein binds only to the full-length COPA and COPA-Δ816–1224, indicating that the WD-repeat domains are essential for recognizing the CT (Fig. 2I). In summary, these results demonstrate that the IBV S protein interacts directly with the WD-repeat domains of COPA via its CT.

### IBV infection does not regulate COPA expression

**Fig 3:**
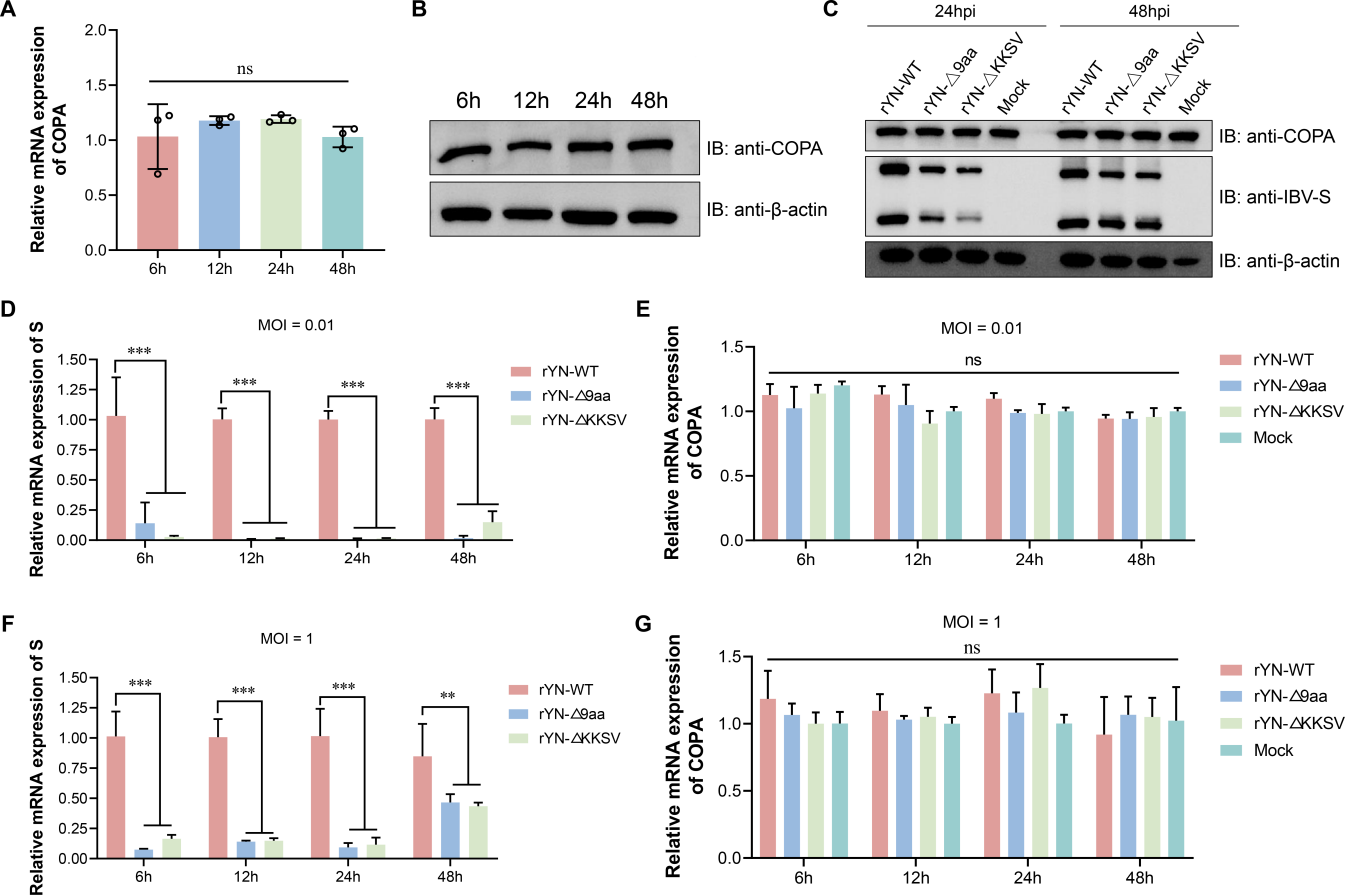
IBV infection does not regulate COPA expression. (**A**) The mRNA expression levels of COPA in control CEK cells at various time points. (**B**) The protein expression levels of COPA in control CEK cells at various time points. (**C**) The protein expression levels of COPA and S protein in cells infected with rIBVs (rYN-WT, rYN-Δ9aa, and rYN-ΔKKSV) at an MOI of 0.01. (**D**) The mRNA expression levels of S protein in cells infected with rIBVs (rYN-WT, rYN-Δ9aa, and rYN-ΔKKSV) at an MOI of 0.01. (**E**) The mRNA expression levels of COPA in cells infected with rIBVs (rYN-WT, rYN-Δ9aa, and rYN-ΔKKSV) at an MOI of 0.01. (**F**) The mRNA expression levels of S protein in cells infected with rIBVs (rYN-WT, rYN-Δ9aa, and rYN-ΔKKSV) at an MOI of 1. (**G**) The mRNA expression levels of COPA in cells infected with rIBVs (rYN-WT, rYN-Δ9aa, and rYN-ΔKKSV) at an MOI of 1.

To investigate whether the S protein of IBV regulates the expression of the host COPA gene, we first assessed the baseline expression levels of COPA in uninfected chicken embryo kidney (CEK) cells over time. qPCR analysis revealed that endogenous COPA mRNA levels remained stable (Fig. 3A). Similarly, Western blot analysis confirmed that COPA protein levels were also steady (Fig. 3B). Next, CEK cells were infected with IBV strains rYN-WT, rYN-Δ9aa, and rYN-ΔKKSV at a multiplicity of infection (MOI) of 0.01. Cells were harvested at 24 and 48 h post-infection, and the protein expression levels of IBV S protein and host COPA were examined. The results indicated that COPA protein levels remained constant regardless of IBV infection or the presence of CT mutations in the S protein (Fig. 3C). The mRNA expression levels were also examined. Cells were infected with IBV strains at an MOI of 0.01 or 1 and harvested at 6, 12, 24, and 48 h post-infection, and the mRNA levels of IBV S protein and host COPA were quantified using RT-qPCR. The results showed that the mRNA levels of the S protein were significantly higher in cells infected with rYN-WT compared to those infected with rYN-Δ9aa and rYN-ΔKKSV (Fig. 3D and F). However, there were no significant differences in COPA mRNA levels between the infected and control groups (Fig. 3E and G). To facilitate accurate interpretation of the data from 6 to 48 h post-infection, we have supplemented our findings with IFA data showing the percentage of virus-infected cells (see Fig. S1). These findings collectively indicate that IBV infection does not regulate the expression of the host COPA gene.

### Knockdown of the COPA enhances S protein trafficking to the plasma membrane

**Fig 4:**
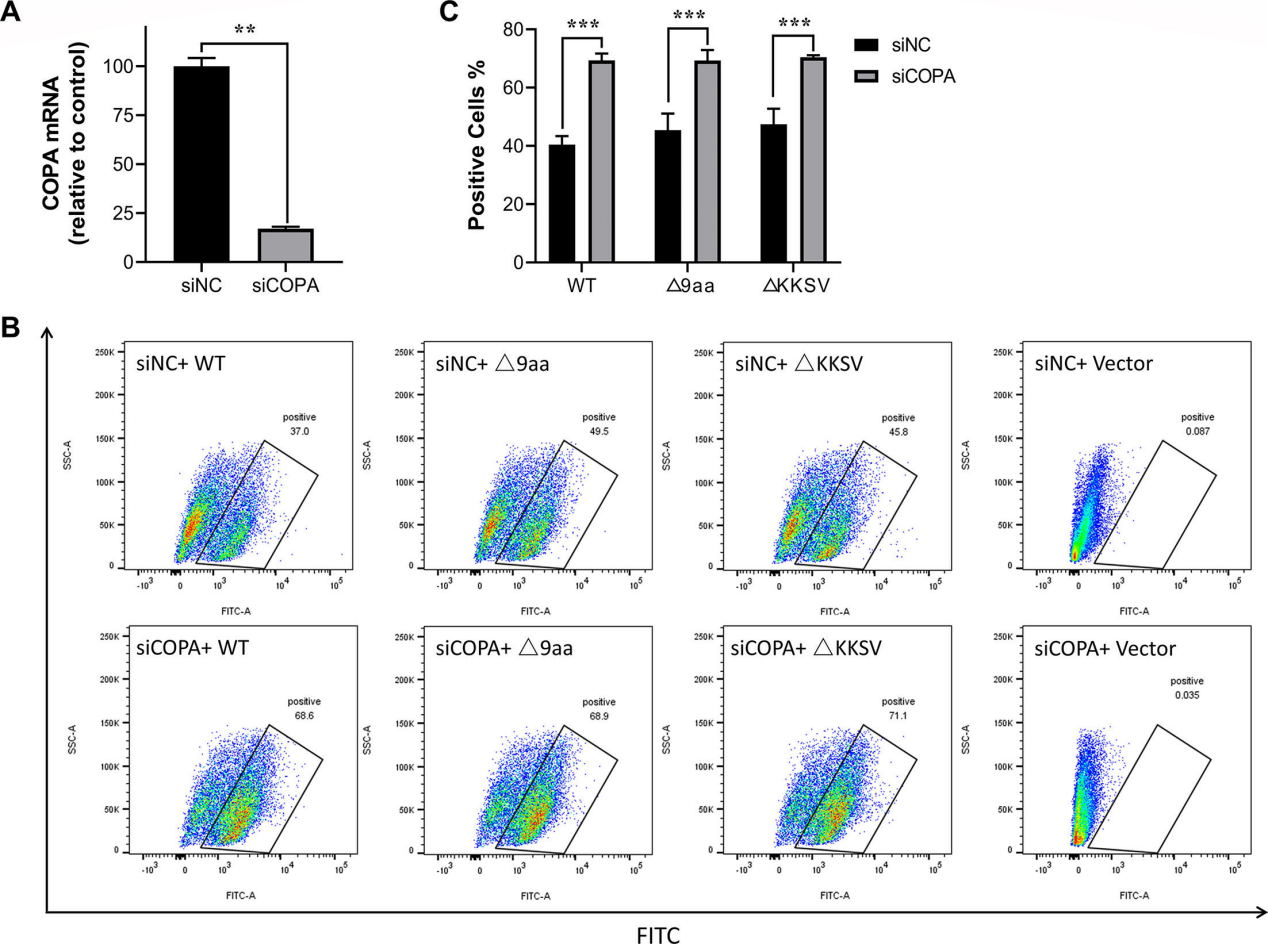
COPA knockdown enhances S protein trafficking to the plasma membrane. (**A**) Interference efficiency assessment. COPA mRNA expression levels were measured by RT-qPCR 24 h post-transfection with siNC or siCOPA. (**B**) Flow cytometric evaluation of the cell surface expression of S proteins. HEK-293T cells were transfected with the siCOPA, 24 h after siRNA transfection, the cells were subsequently transfected with the corresponding plasmids (S-WT, S-Δ9aa, and S-ΔKKSV). At 36 h post-plasmid transfection, non-permeabilized cells were immunolabeled with anti-Flag primary antibody and Alexa Fluor 488 (AF488)-conjugated secondary antibody. Surface S protein expression was quantified as the percentage of AF488-positive cells using a BD FACSCanto II flow cytometer and analyzed with FlowJo software. (**C**) Quantitative analysis of surface S protein expression. Data represent mean ± SEM from three independent experiments. Statistical significance was determined using two-way ANOVA. *P < 0.05; **P < 0.01; and ***P < 0.001.

Our previous studies have demonstrated that the deletion of the KKSV motif in the CT of the S protein significantly reduces its transport to the ERGIC for assembly, resulting in increased escape of the S protein to the plasma membrane. To further elucidate the role of COPA in S protein trafficking, we first transfected cells with siCOPA to knock down COPA expression, using siNC as a negative control. As shown in Fig. 4A, the knockdown efficiency reached 80%. After 24 h of interference, cells were transfected with either wild-type IBV S protein (S-WT) or its mutants, with an empty vector serving as a control. After an additional 36 h, cells were harvested, and the expression of S protein on the cell surface was analyzed by flow cytometry. As illustrated in Fig. 4B, in the siNC group, S-Δ9aa and S-ΔKKSV exhibited significantly higher plasma membrane expression compared to S-WT, consistent with our previous findings and indicating that deletion of the KKSV motif in the CT enhances S protein escape to the plasma membrane. In the vector group (pRK5-Flag), no difference was observed between siNC and siCOPA treatments. However, in groups transfected with S protein and its mutants, siCOPA treatment significantly increased S protein expression on the plasma membrane compared to siNC treatment (Fig. 4C). This effect was particularly pronounced in the S-WT group, where plasma membrane expression increased by approximately 30%. To quantitatively assess this differential response across mutants, we calculated the slope (*K*) of the response curves, where the absolute *K* value correlates with the magnitude of COPA knockdown effects. As demonstrated in the Fig. S2, the WT-S exhibits a significantly steeper slope, confirming its heightened sensitivity to siCOPA. These results suggest that COPA plays a crucial role in the retrograde transport of S protein to the ERGIC. Interference with COPA expression leads to increased S protein escape to the plasma membrane.

### Arf1-dependent COPI transportation is critical for IBV infection

**Fig 5:**
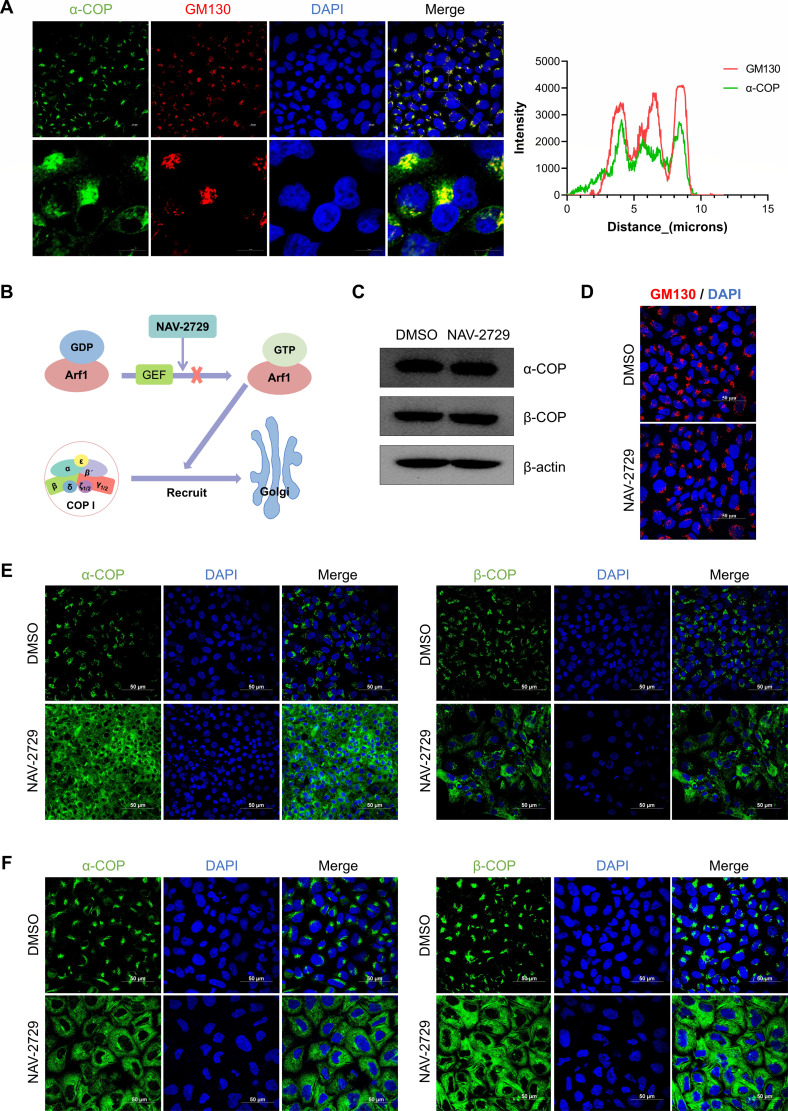
NAV-2729 inhibits the Arf1-dependent COPI transport. (**A**) Immunofluorescence analysis of COPI vesicle interactions with the Golgi apparatus. CEK cells were fixed and immunolabeled with anti-COPA and anti-GM130 antibodies, followed by Alexa Fluor 488- and 555-conjugated secondary antibodies, respectively. Nuclei were stained with DAPI. (**B**) Schematic overview of NAV-2729’s mechanism of action. NAV-2729 disrupts COPI recruitment to the Golgi membrane by inhibiting Arf1 nucleotide exchange. (**C**) Impact of NAV-2729 on COPI expression. CEK cells were treated with DMSO or NAV-2729 for 48 h, lysed, and analyzed by immunoblotting using the indicated antibodies. β-actin was used as a loading control. (**D**) Effect of NAV-2729 on the Golgi apparatus. CEK cells were treated with DMSO or NAV-2729 for 48 h, fixed, and immunolabeled with anti-GM130 antibodies, followed by Alexa Fluor 555-conjugated secondary antibodies. Nuclei were stained with DAPI. (**E**) Effect of NAV-2729 on COPI recruitment to the Golgi membrane in CEK cells. CEK cells treated with DMSO or NAV-2729 for 48 h were fixed and immunolabeled with anti-COPA/COPB and anti-GM130 antibodies, followed by Alexa Fluor 488- and 555-conjugated secondary antibodies, respectively. Nuclei were stained with DAPI. (**F**) Effect of NAV-2729 on COPI recruitment to the Golgi membrane in BHK-21 cells.

COPI vesicles primarily mediate retrograde transport within the Golgi apparatus, ensuring its normal function and structural maintenance. We initially confirmed the endogenous localization of COPI vesicles predominantly in the Golgi apparatus through IFA, using the COPA subunit as a marker for COPI vesicles and GM130 for the Golgi apparatus (Fig. 5A). To further investigate the requirement of COPI vesicle trafficking for IBV infection, we disrupted COPI recruitment to the Golgi membrane by inhibiting the small guanosine tri-phosphatase (GTPase) ADP-ribosylation factor 1 (Arf1), thereby interfering with COPI vesicle transport. Arf1, a small GTPase protein, is inactive in its GDP-bound state. Arf1 activation requires GDP-to-GTP exchange, catalyzed by guanine nucleotide exchange factors (GEFs). In its GTP-bound state, Arf1 simultaneously binds to the Golgi membrane and the COPI, recruiting COPI to the membrane (24, 25). The chemical inhibitor NAV-2729 prevents Arf1 binding to the Golgi membrane and subsequent COPI recruitment by inhibiting nucleotide exchange on Arf1 (26, 27) (Fig. 5B). To evaluate the inhibitor’s effects, we first assessed the levels of individual COPI subunits (α, β) by Western blot, which remained unaffected by inhibitor treatment (Fig. 5C). Additionally, Golgi structure integrity was confirmed through GM130 staining (Fig. 5D). We then evaluated the impact of NAV-2729 treatment on COPI vesicles in both CEK and BHK-21 cells. In DMSO-treated control cells, α-COPI and β-COPI were concentrated near the Golgi apparatus, whereas inhibitor treatment resulted in diffuse cytoplasmic distribution of COPI, preventing its localization to the Golgi (Fig. 5E and F). These results confirm that NAV-2729 inhibitor treatment prevents COPI localization to the Golgi membrane without affecting COPI expression levels.

**Fig 6:**
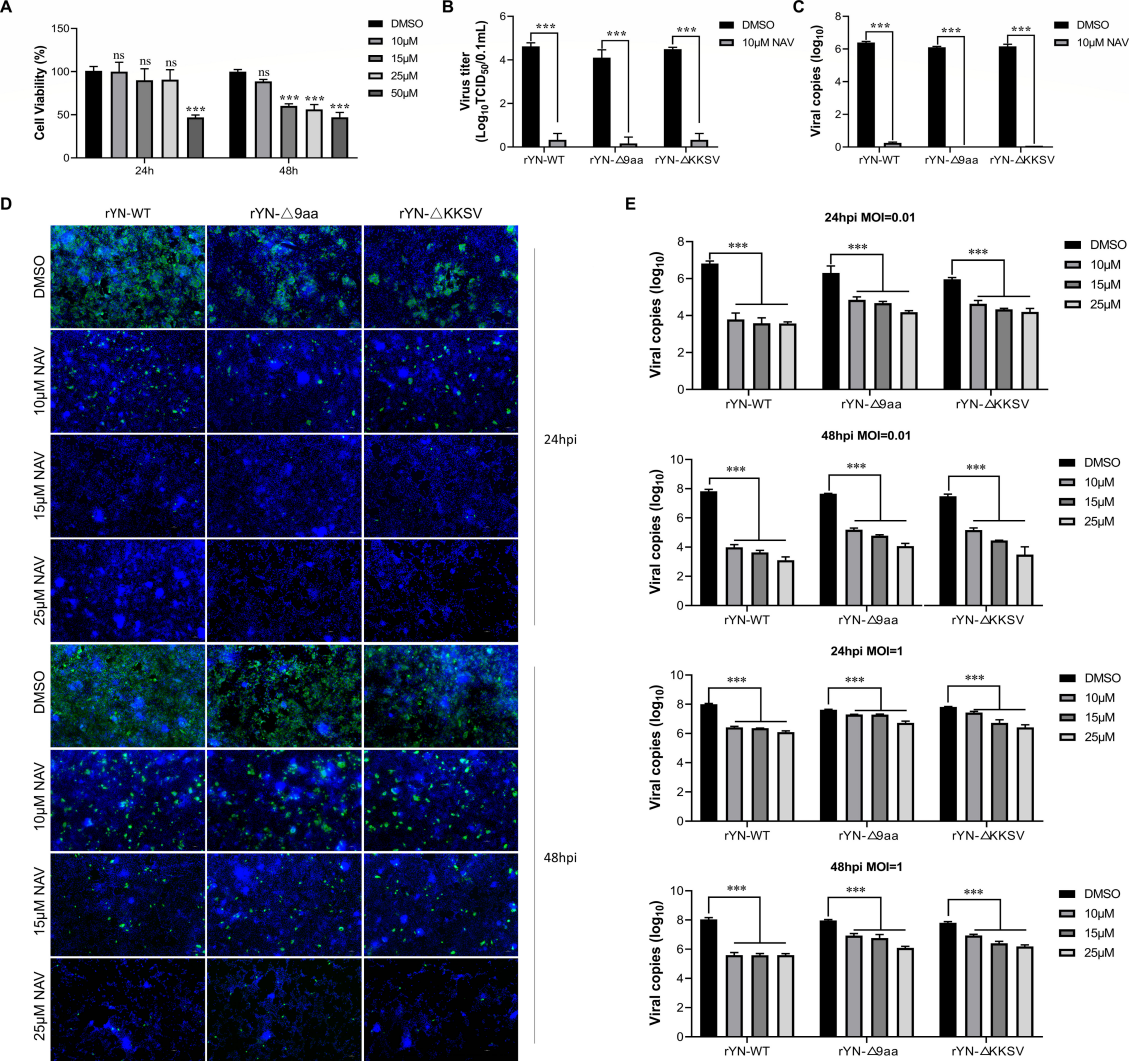
NAV-2729 impairs the production of IBV progeny virions. (**A**) Impact of NAV-2729 on cell viability. CEK cells were treated with varying concentrations of NAV-2729 for 24–48 h. Cell viability was assessed using the CCK-8 assay. (**B–E**) Effect of NAV-2729 on IBV proliferation. CEK cells were pre-treated with 10 µM NAV-2729 for 1 h prior to infection with recombinant IBVs (rIBVs) at an MOI of 0.01. Post-infection, treatment with 10 µM NAV-2729 continued. After 48 h, cell supernatants were collected to determine viral titers (**B**) and viral RNA copy numbers (**C**). Additionally, cells treated with NAV-2729 6 h post-infection at an MOI of 0.01 were immunolabeled with anti-IBV-N antibodies, followed by Alexa Fluor 488-conjugated secondary antibodies. Nuclei were stained with DAPI (**D**), and viral particle copy numbers in the supernatant were quantified (**E**).

We evaluated the impact of different concentrations of NAV-2729 on cell viability using the CCK8 assay. Results showed that after 24 h of treatment, only 50 µM significantly affected cell viability; after 48 h, all concentrations except 10 µM significantly reduced cell viability (Fig. 6A). To further investigate the effect of NAV-2729 on IBV infection, the cells were pre-treated with 10 µM NAV-2729 for 1 h prior to infection with IBV at an MOI of 0.01. Treatment with 10 µM inhibitor continued post-infection. After 48 h, cell supernatants were collected to determine viral titers and genomic copy numbers. Results demonstrated that the NAV-treated group had virtually undetectable viral copy numbers, and the treated virus lost infectivity (Fig. 6B and C). We also examined the effect of NAV inhibitor on viral attachment. As shown in Fig. S3, pretreatment with NAV inhibitor did not affect the attachment capability of any viral strains tested. Subsequently, we treated cells with NAV-2729 6 h post-infection (after one viral replication cycle) and assessed viral infection by IFA. As shown in Fig. 6D, increasing NAV concentrations progressively reduced infection rates in a dose-dependent manner, particularly for the rYN-WT strain. We also infected cells with rYN-WT and mutant strains (rYN-Δ9aa and rYN-ΔKKSV) at MOIs of 0.01 and 1, and quantified supernatant viral genomic copy numbers. Results confirmed that NAV treatment significantly reduced IBV replication (Fig. 6E), with a more pronounced effect on the rYN-WT strain. To systematically evaluate the differential responses across viral strains under various treatment conditions, we employed two analytical approaches. First, we calculated interaction *P* values, which were consistently below 0.0001 across all groups, indicating statistically significant strain-specific responses to different treatments. Second, we quantified sensitivity through slope (*K*) analysis of the treatment-response curves. As illustrated in Fig. S4, the WT strain exhibits the steepest slope (highest absolute value), confirming its superior sensitivity to NAV inhibitor treatment. Collectively, these findings demonstrate that inhibition of COPI-mediated transport by NAV-2729 impairs the production of IBV progeny virions, underscoring the importance of COPI-dependent trafficking in the IBV life cycle.

### Inhibition of COPI-mediated transport enhances S protein trafficking to the plasma membrane

**Fig 7:**
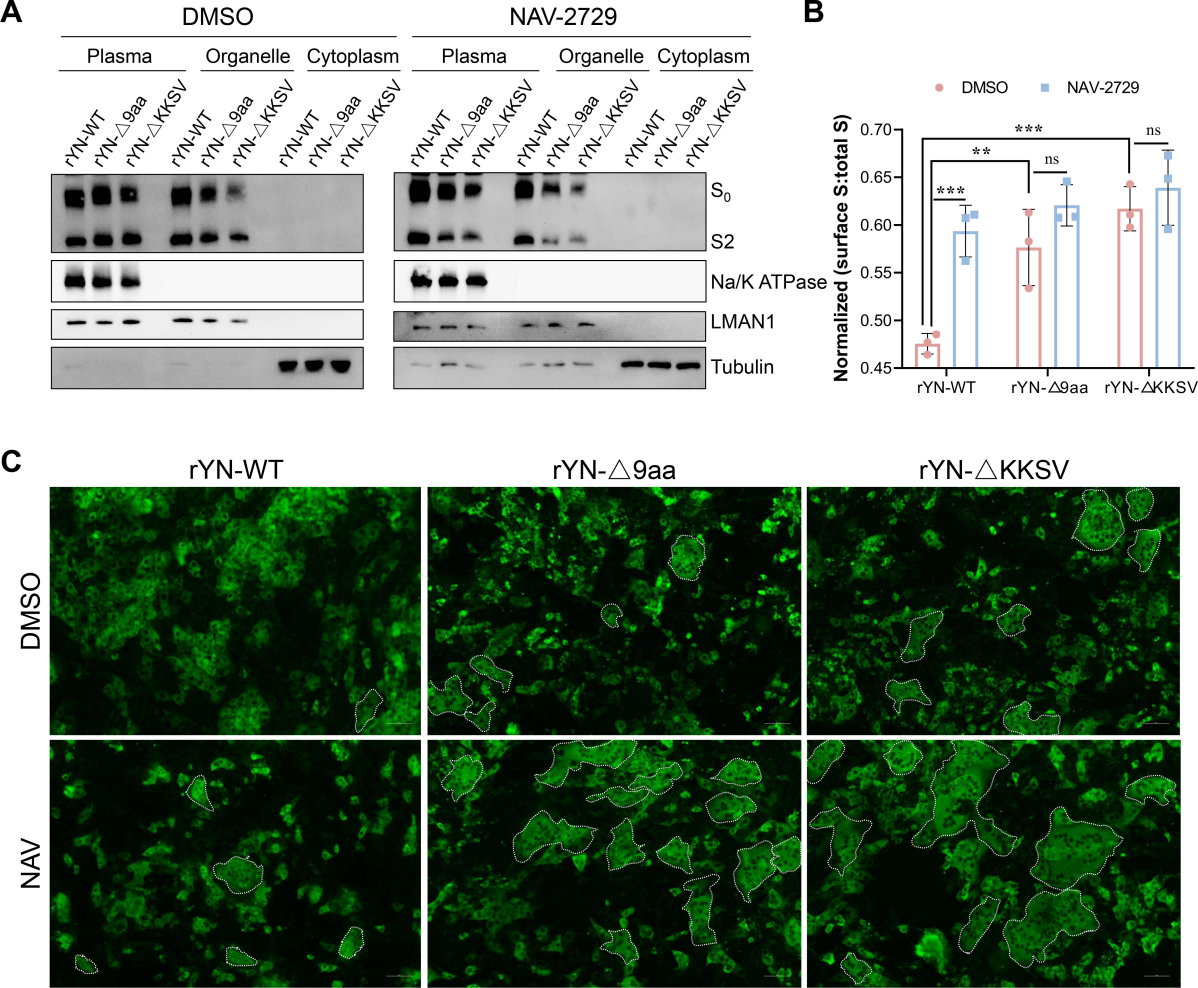
Inhibition of COPI-mediated transport enhances S protein trafficking to the plasma membrane. (**A**) Western blot analysis of cell surface S protein expression. CEK cells were infected with IBV at an MOI of 1, treated with NAV-2729 6 h post-infection, and harvested at 36 h post-infection. Proteins from various cellular fractions were isolated and analyzed. Na/K ATPase, LMAN1, and tubulin were used as loading controls for the plasma membrane, total membrane, and cytoplasmic fractions, respectively. (**B**) Quantification of S protein expression in the cell surface. S protein levels were calculated as the sum of the amounts of full-length S0 and cleaved S2 products, whereby the amount of plasma membrane S indicated cell surface expression and total S reflected the total amount of S protein across all cellular fractions. (**C**) Evaluation of syncytia formation caused by IBV infection. CEK cells were immunolabeled with anti-IBV-N antibodies, followed by Alexa Fluor 488-conjugated secondary antibodies. Syncytia are indicated by circled regions.

By the above-described results, we have demonstrated that the IBV S protein interacts with COPI via the KKSV motif in its CT. Disruption of COPI expression resulted in increased S protein trafficking to the plasma membrane. To further elucidate whether this effect is specifically related to COPI-mediated transport rather than merely weakened COPI binding, we first performed membrane fractionation experiments to specifically detect S protein expression on the plasma membrane. CEK cells were infected with rYN-WT and mutant strains (rYN-Δ9aa and rYN-ΔKKSV), then treated with either DMSO or NAV-2729 for 36 h before fractionation. Results showed that S protein was primarily distributed between the plasma membrane and cellular organelles, with negligible cytoplasmic expression. In DMSO-treated cells, rYN-WT exhibited comparable S protein levels in the plasma membrane and organelle fractions, while mutant strains rYN-Δ9aa and rYN-ΔKKSV showed significantly higher S protein levels in the plasma membrane fraction. NAV treatment resulted in increased plasma membrane localization of S protein for all viral strains, with the rYN-WT group displaying particularly significant differences compared to the DMSO control group (Fig. 7A and B), indicating that COPI transport inhibition leads to enhanced S protein escape to the plasma membrane. Like other CoVs, IBV assembles and buds intracellularly. Consequently, S proteins that reach the plasma membrane do not participate in virion formation. However, these surface-expressed S proteins can induce cell-cell fusion, forming syncytia and facilitating viral spread. As shown in Fig. 7C, we observed syncytia formation in all IBV-infected cells. Notably, the cells infected with the CT mutants rYN-Δ9aa and rYN-ΔKKSV exhibited more extensive syncytia formation compared to rYN-WT. Moreover, NAV-2729 treatment resulted in larger and more numerous syncytia relative to the DMSO control across all viral strains. These results suggest that inhibition of COPI-mediated transport prevents efficient trafficking of S proteins to the ERGIC for virion assembly, thereby impacting the production of IBV progeny particles. Concurrently, the increased presence of S proteins at the plasma membrane promotes cell-cell fusion.

## DISCUSSION

CoVs are characterized by their unique assembly and budding process, which occurs in the ERGIC, followed by exocytosis. The advantages of this intracellular assembly remain elusive. CoVs primarily contain three membrane proteins: S, M, and E. To ensure their accumulation in the ERGIC, these viral membrane proteins typically possess signals that direct them to the virus assembly site (28). For instance, the E protein contains a Golgi-targeting signal within its CT (29), while the M protein has targeting motifs in its CT that direct it to the assembly site (30–32). For the S protein, the ERRS motif in its CT is likely to play a crucial role in facilitating its retrograde transport from the Golgi to the ERGIC (33, 34). The ERRS motif varies among different coronavirus genera: most α- and β-CoVs exhibit a KxHxx motif, γ-CoVs display a KKxx motif, and δ-CoVs present a KxKxx motif (35). However, direct evidence demonstrating whether these diverse ERRS motifs function equivalently in retaining the S protein in the ERGIC for assembly is lacking. Recent studies on SARS-CoV-2 suggest that its KxHxx motif serves as a suboptimal ER retrieval signal (10, 11). Our previous research has shown that the KKSV motif in the IBV S protein is critical for its retention in the ERGIC (22). Deletion or mutation of this KKSV motif results in decreased localization of the S protein in the ERGIC and increased expression on the plasma membrane. These findings indicate that the KKSV motif in the CT of the IBV S protein functions as a canonical ERRS, responsible for intracellular retention of the S protein during viral assembly.

Due to the absence of organelles found in living cells, viruses must hijack host cell membrane structures and associated pathways to facilitate their life cycle. Our study focuses on elucidating how the KKSV motif in the CT of the IBV S protein co-opts host proteins for its transport to the ERGIC for assembly. COPI-coated vesicles are responsible for retrograde transport within cells, and COPI specifically recognizes di-lysine sequences in cargo proteins (12, 36). We therefore hypothesized that COPI-coated vesicles might be involved in S protein trafficking. Initially, we confirmed the colocalization and interaction between the IBV S protein and various COPI subunits using IFA and Co-IP (Fig. 1). Furthermore, GST pull-down experiments demonstrated that COPI directly binds to the CT of the S protein (Fig. 2F). A recent study on SARS-CoV-2 (10), using affinity chromatography, also showed direct binding between COPI and the S protein’s CT, suggesting that this domain is crucial for COPI interact with the S protein. We further demonstrated that the KKSV motif in the CT is critical for COPI recognition of the S protein. Deletion of either the 9 aa or the KKSV motif significantly weakened the interaction between the S protein and COPI (Fig. 2). Moreover, we found that the WD repeat domain of α-COPI plays a key role in recognizing the KKSV motif in the S protein. The WD repeat domain, characterized by alternating tryptophan (Trp, W) and aspartic acid (Asp, D) residues, is found in various proteins involved in intracellular transport and signal transduction. Previous studies using yeast two-hybrid assays have shown that the WD40 domains of α- and β′-COP recognize different di-basic signals: α-COP WD40 recognizes KKxx signals, while β′-COP WD40 recognizes KxKxx signals (23). This differential recognition suggests a mechanism for the specific interaction between COPI and the IBV S protein’s KKSV motif.

Accumulating evidence suggests that COPI plays a crucial role in the life cycles of various viruses. The mechanisms by which COPI influences viral processes can be categorized into two main aspects. First, COPI vesicle transport pathways may shuttle cargo essential for viral replication, including host and viral proteins. For instance, human papillomavirus utilizes COPI for transport during viral entry (37). COPI vesicles are also implicated in the early stages of gene expression and later stages of infectious virus assembly and egress in Hazara nairovirus (38). Additionally, lymphocytic choriomeningitis arenavirus requires cellular COPI for efficient virion production (39). Second, key COPI components may be co-opted by viruses to perform functions unrelated to vesicular transport. For example, studies have shown that the NS1 protein of porcine parvovirus interacts with ε-COPI to modulate cellular IFN-β production, thereby affecting viral replication (40). Inhibition of COPI expression has been observed to cause vesicular stomatitis virus internalization arrest and secondary defects in viral gene expression (41). Furthermore, interference with β-COP has been shown to inhibit the formation of Drosophila C virus (DCV) replication compartments, consequently suppressing viral replication (42). However, the impact of COPI inhibition or disruption of its transport function on the life cycle of IBV and other CoVs remains unexplored. Our research demonstrates that IBV infection, including its mutant strains, does not affect COPI expression levels (Fig. 3). This suggests that COPI treats IBV viral proteins as standard cargo, similar to escaped ER proteins, performing its transport function. Current research on the interaction between coronavirus S proteins and COPI has primarily focused on their basic interaction, with no definitive studies demonstrating the effects of COPI inhibition on S protein trafficking. Our study reveals that knockdown of the COPA subunit significantly increases plasma membrane expression of independently expressed S protein, suggesting that COPI plays a crucial role in S protein transport (Fig. 4). Due to the inability to transfect siRNA in the IBV-susceptible CEK cells, we employed COPI transport inhibitors to investigate S protein trafficking during infection (Fig. 5). Results indicate that COPI transport inhibition leads to increased S protein expression on the plasma membrane, inducing cell-cell fusion and syncytia formation in adjacent cells (Fig. 7). Our findings align with a recent SARS-CoV-2 study (21), which demonstrated that COPI sorting inhibitors enhance S protein exposure on host cell surfaces. When we inhibited COPI transport function prior to viral infection, we observed that IBV failed to form infectious progeny virions (Fig. 6), indicating that COPI-mediated transport plays a critical role in IBV particle assembly.

In addition to host COPI-mediated regulation of S protein trafficking, research indicates that viral M and E proteins also modulate S protein intracellular transport and processing during infection (43). The E protein induces S protein retention in the ERGIC by regulating cellular secretory pathways (44), while the M protein recruits S protein to the ERGIC by recognizing its CT ERRS motif. Interestingly, mutations in the ERRS motif do not prevent M-S interactions. However, S proteins with intact ERRS motifs undergo repeated cycling through the ER-Golgi system, providing ample opportunities for interaction with M proteins (9). This phenomenon may explain why, under COPI transport inhibition during infection, the rYN-WT group did not exhibit significantly higher plasma membrane expression or larger syncytia compared to the mutant groups (rYN-Δ9aa and rYN-ΔKKSV).

The transport of S proteins to the cell surface has significant implications for coronavirus vaccine design. Current mRNA or adenovirus vector-based vaccines rely on the expression of full-length S proteins (45, 46). Only cell surface-exposed S is able to be directly recognized by the B-cell receptor, which is the trigger step for B cell activation after S mRNA vaccination (21, 45, 47). By modifying the S protein’s CT or incorporating COPI transport inhibitors could enhance S protein expression on the plasma membrane, thereby promoting antigen recognition by the host immune system and potentially eliciting a more robust immune response. Such a strategy might potentially be combined with the use of S mRNA vaccine in clinics.

**Fig 8:**
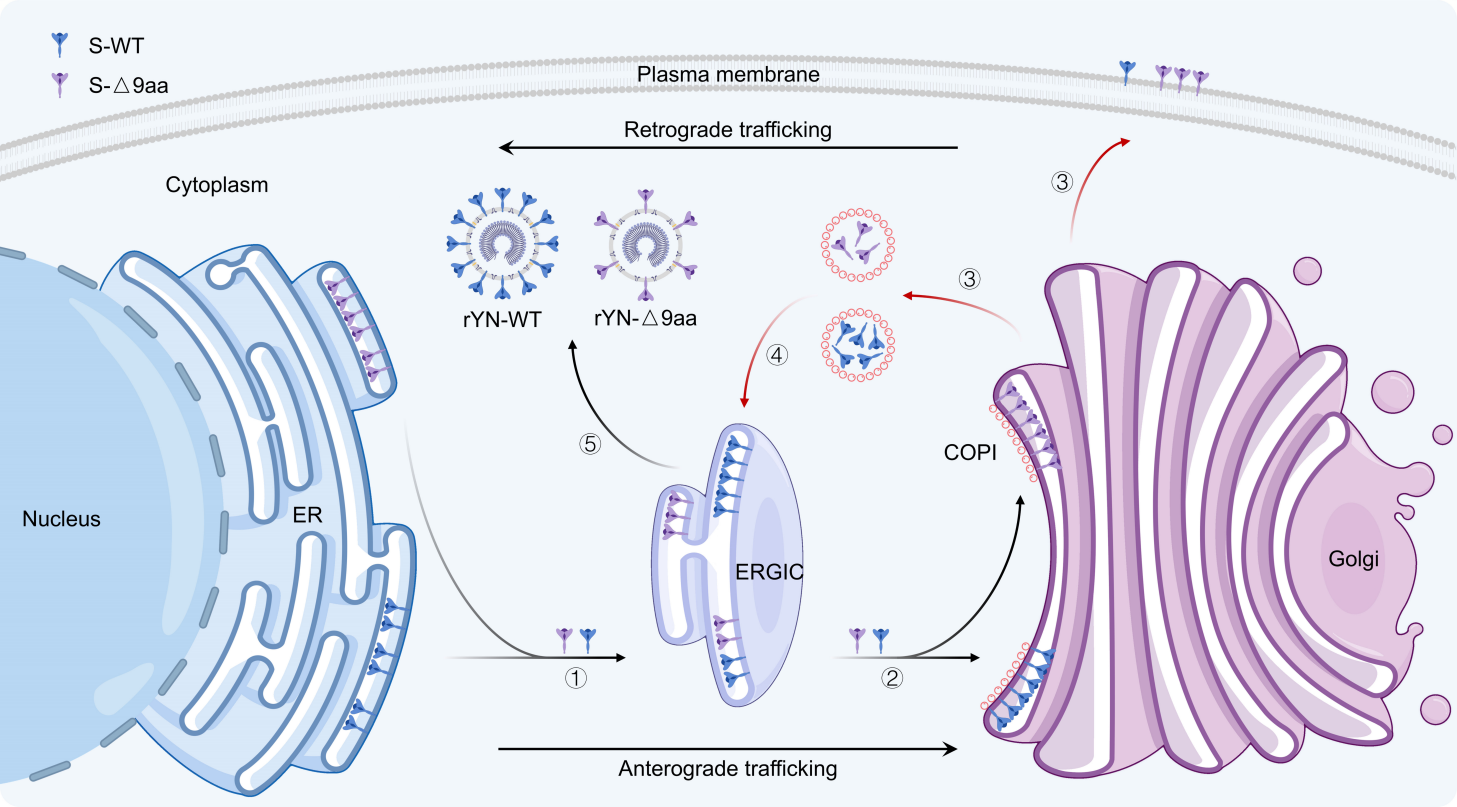
Graphical study summary. The IBV S protein is recognized by COPI through its KKSV motif in the cytoplasmic tail. Deletion of either the 9 aa or the KKSV motif results in reduced binding to COPI, leading to increased escape of the S protein to the plasma membrane.

In summary, our research demonstrates that COPI is a crucial protein responsible for trafficking the IBV S protein to the ERGIC for viral assembly. The IBV S protein is recognized by COPI through its KKSV motif in the CT. Deletion of either the 9 aa or the KKSV motif results in reduced binding to COPI, leading to the increased escape of the S protein to the plasma membrane (Fig. 8). Our findings not only advance our understanding of IBV S protein trafficking mechanisms and enhance our understanding of viral replication mechanisms, but also provide valuable insights for developing more effective vaccine strategies.

## MATERIALS AND METHODS

### Viruses and cells

Recombinant IBV strains rYN-WT, rYN-Δ9aa, and rYN-ΔKKSV were rescued and preserved in our laboratory, and propagated in the allantoic fluid of 10-day-old SPF chicken embryos. BHK-21 and HEK-293T cell lines were cultured in Dulbecco’s modified Eagle’s medium (Gibco, USA) supplemented with 10% fetal bovine serum and 1% penicillin-streptomycin. CEK cells were isolated from 18-day-old SPF chicken embryos. All cell cultures were maintained in a humidified incubator at 37°C with 5% CO_₂_.

### Plasmid construction and transfection

The full-length S gene was amplified from YN cDNA (GenBank JF893452.2). The S-WT and its mutants (S-Δ9aa and S-ΔKKSV) were cloned into the pRK5-Flag vector. Each subunit of COPI, including α, β, β′, γ1/2, δ, ε, and ζ, was cloned into the PCAGGS-HA vector. For the GST-pulldown assays, the plasmid GST-CT, which contains the 38 aa of the S protein CT, was cloned into the prokaryotic expression vector pGEX-6P-1 for individual expression. Mutants GST-CT-Δ9aa and GST-CT-ΔKKSV were generated by site-directed mutagenesis based on GST-CT. COPA-WT, COPA-Δ1–318, COPA-Δ324–774, and COPA-Δ816–1224 were cloned into the pET-28a-His vector.

For transfections, the cells were transfected with the aforementioned plasmids using StarFect transfection reagent (GenStar, China) according to the manufacturer’s instructions. Briefly, plasmids and StarFect were diluted in Opti-MEM (Gibco, USA) at a 1:3 ratio and incubated together for 15 min. The plasmid/StarFect complexes were then incubated with cells in a cell culture incubator.

### Indirect IFA and confocal microscopy

Cell samples were collected at predetermined time points post-transfection or -infection and fixed using Immunol Staining Fix Solution (Beyotime Biotechnology, China). This was followed by permeabilization with an Immunostaining Permeabilization Buffer containing Triton X-100 (Beyotime Biotechnology) and subsequent blocking with an Immunol Staining Blocking Buffer (Beyotime Biotechnology). The cells were then incubated with specific primary antibodies at 4°C for 12 h. For staining, Alexa Fluor 488-conjugated anti-mouse IgG (H + L) and/or Alexa Fluor 555-conjugated anti-rabbit IgG (H + L) (Cell Signaling Technology, USA) were applied at room temperature in the dark for 1 h. Nuclei were stained with DAPI (Sigma-Aldrich, USA) at room temperature for 10 min. Subsequently, the cells were washed five times with PBST (phosphate-buffered saline [PBS] with Tween 20), with each wash lasting 5 min. Finally, the cells were observed and imaged using a Nikon A1 fluorescence microscope (Nikon, Tokyo, Japan). Colocalization analysis was performed using the Fiji ImageJ software. PCC is commonly employed to assess the spatial colocalization of two fluorescently labeled molecules or structures within a cell. The PCC values range from −1 to +1, where +1 indicates a perfect positive correlation (complete colocalization), 0 indicates no correlation (random distribution), and −1 indicates a perfect negative correlation.

### Co-IP assay

Following transfection with the constructed plasmids or infection with recombinant viruses, cell pellets were collected at designated time points. Cells were lysed in 1 mL of Cell Lysis Buffer for Western and IP (Beyotime Biotechnology, Shanghai, China). The lysates were centrifuged at 14,000 *× g* for 10 min, and the supernatants were pre-cleared with mouse IgG agarose (Beyotime Biotechnology, Shanghai, China) for 2 h and then incubated overnight at 4°C with either anti-Flag M2 affinity gel (Sigma-Aldrich, USA) or HA-conjugated protein A/G magnetic beads (Santa Cruz Biotechnology, USA). For endogenous Co-IP, lysates were incubated overnight at 4°C with protein A/G magnetic beads pre-coupled with anti-COPA antibody (Mouse, Santa Cruz Biotechnology, USA). The resulting immunoprecipitates and cell lysates were further analyzed by Western blotting.

### GST pull-down assay

*Escherichia coli* BL21-DE3 strains (TransGen, Beijing, China) containing pGEX-6P-1, pGEX-GST-CT, pGEX-GST-CT-Δ9aa, or pGEX-GST-CT-ΔKKSV were grown in lysogeny broth medium at 37°C for 4–6 h. Isopropyl β-d-1-thiogalactopyranoside (IPTG) was then added to the cultures to a final concentration of 1 mM, and the cells were further incubated at 16°C for 14–16 h. The cell pellets were lysed on ice using an ultrasonic cell disruptor. The lysates were clarified by centrifugation and incubated with glutathione Sepharose beads (GE Healthcare, Uppsala, Sweden) at room temperature for 2 h. After washing three times with PBS, the beads were incubated overnight at 4°C with lysates from cells transfected with PCAGGS-HA-COPA or bacterial lysates containing pET-28a-COPA-WT, pET-28a-COPA-Δ1–318, pET-28a-COPA-Δ324–774, or pET-28a-COPA-Δ816–1224. The beads were washed three times with PBS and eluted with Tris-Cl (50 mM; pH 8.0) containing 20 mM reduced glutathione. The bound proteins were then analyzed by Western blot.

### Real-time quantitative PCR

Total RNA was extracted using the Total RNA Isolation Kit (Magen, Beijing, China) and subsequently reverse-transcribed into cDNA. Real-time quantitative PCR (RT-qPCR) was performed on the cDNA using the M5 HiPer Real-Time PCR Super Mix (Mei5bio, Beijing, China). The mRNA levels of target genes in each sample were normalized to the mRNA levels of glyceraldehyde-3-phosphate dehydrogenase (GAPDH). Each experiment was independently repeated three times.

### siRNA transfection

HEK-293T cells were transfected with the specified siRNA using RNAi-Mate (GenePharma, Shanghai, China) according to the manufacturer’s instructions. The siRNAs used in this study were designed and synthesized by GenePharma (Shanghai, Beijing). Twenty-four hours after siRNA transfection, the cells were subsequently transfected with the corresponding plasmids (S-WT, S-Δ9aa, and S-ΔKKSV).

### Flow cytometric assessment of cell surface S protein levels

At 36 h post-transfection, the cells were washed three times with ice-cold PBS through resuspension and centrifugation at 500 × *g* for 5 min at 4°C. The supernatant was then discarded, and 10^6^ cells were resuspended in PBS containing a 1:1,000 dilution of anti-Flag antibody (CST) and incubated at 4°C for 1 h. Subsequently, the cells were washed three times with ice-cold PBS and then incubated on ice in the dark for 1 h with PBS containing FITC-conjugated goat anti-rabbit immunoglobulin G antibodies (diluted 1:500). After three additional washes, the cells were resuspended in 100 µL of cold PBS and strained through a 100 µm filter before being analyzed using a BD FACSCanto II flow cytometer (BD Biosciences). Data were processed using FlowJo software.

### Cytotoxicity and drug treatment

The cells were seeded in 96-well plates and allowed to grow for 12 h before being treated with varying concentrations of the NAV-2729 inhibitor (Sigma-Aldrich, USA) for 24–48 h. Cell viability was assessed using the Cell Counting Kit-8 (Beyotime Biotechnology, Shanghai, China) according to the manufacturer’s instructions. Absorbance was measured at 450 nm. Each experiment was independently repeated three times. NAV-2729 was dissolved in dimethyl sulfoxide (DMSO) (Beyotime Biotechnology, Shanghai, China) for use.

### Plasma membrane and cytoplasmic protein extraction

Cells were seeded in a 10-cm culture dish. At 24 h post-seeding, the cells were infected with IBV at an MOI of 1. A cell membrane and cytoplasmic protein extraction kit (Invent) was used to analyze S protein expression at the cell membrane. Briefly, the cells were harvested and washed twice with precooled PBS. After discarding the supernatant, the cells were resuspended in 500 µL of Buffer A and incubated on ice for 10 min. The cell suspension was then transferred to a centrifuge tube column casing and centrifuged at 16,000 × *g* for 30 s. The column was removed, and the sediment was vigorously vortexed, before being centrifuged at 700 × *g* for 1 min. The supernatant containing the cytoplasmic fraction was transferred to a new 1.5 mL centrifuge tube and further centrifuged at 16,000 × *g* for 10 min at 4°C. The precipitate was resuspended in 200 µL of Buffer B and centrifuged at 4°C and 7,800 × *g* for 5 min, yielding the organelle fraction. The supernatant was then mixed with 1.6 mL PBS and centrifuged at 16,000 × *g* for 30 min. The final precipitate contained the plasma membrane fraction. The protein composition of the separated fractions (i.e., cytoplasmic, organelle, and plasma membrane) was analyzed by western blotting.

### Statistical analysis

All data were analyzed with GraphPad Prism software version 5.0 (GraphPad Software Inc., San Diego, CA, USA). Student’s *t* test was used to determine the significance of differences between two groups, while one-way and two-way analyses of variance were for multiple group comparisons. *P* values < 0.05 were used as a measure of statistical significance, with ns, not significant; *, *P* < 0.05; **, *P* < 0.01; and ***, *P* < 0.001 indicated in the figure captions.

## Data Availability

All data in this study are presented here as main and supplemental figures.

## SUPPLEMENTAL MATERIAL

Supplemental figures jvi.02164-24-s0001.pdf Fig. S1 to S4.

ASM does not own the copyrights to Supplemental Material that may be linked to, or accessed through, an article. The authors have granted ASM a non-exclusive, world-wide license to publish the Supplemental Material files. Please contact the corresponding author directly for reuse.
