# Supplementary material for: The cytoplasmic tail of IBV spike mediates intracellular retention via interaction with COPI-coated vesicles in retrograde trafficking
Source: J Virol. 2025 Jan 22;99(2):e02164-24. doi: 10.1128/jvi.02164-24 (PMC11852926; doi:10.1128/jvi.02164-24)
Supplement: Supplemental figures — Fig. S1 to S4. [file jvi.02164-24-s0001.pdf]

Supplemental figures

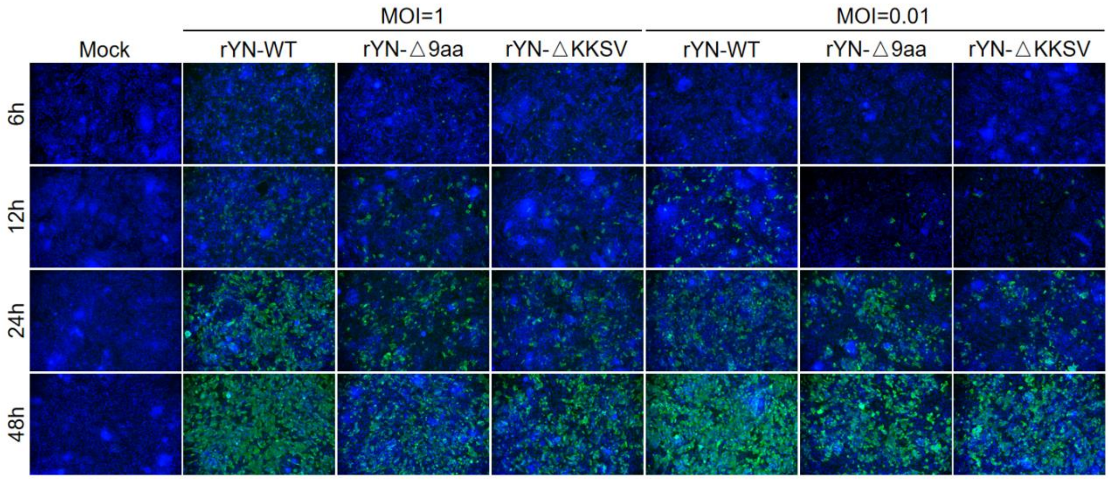

FIG S1 Immunofluorescence staining of IBV-infected CEK cells. CEK cells infected with recombinant IBVs at an MOI of 0.01 or 1 were fixed at 6, 12, 24, 48 hours post-transfection for IFA analysis, with the IBV N protein as visualized in green.

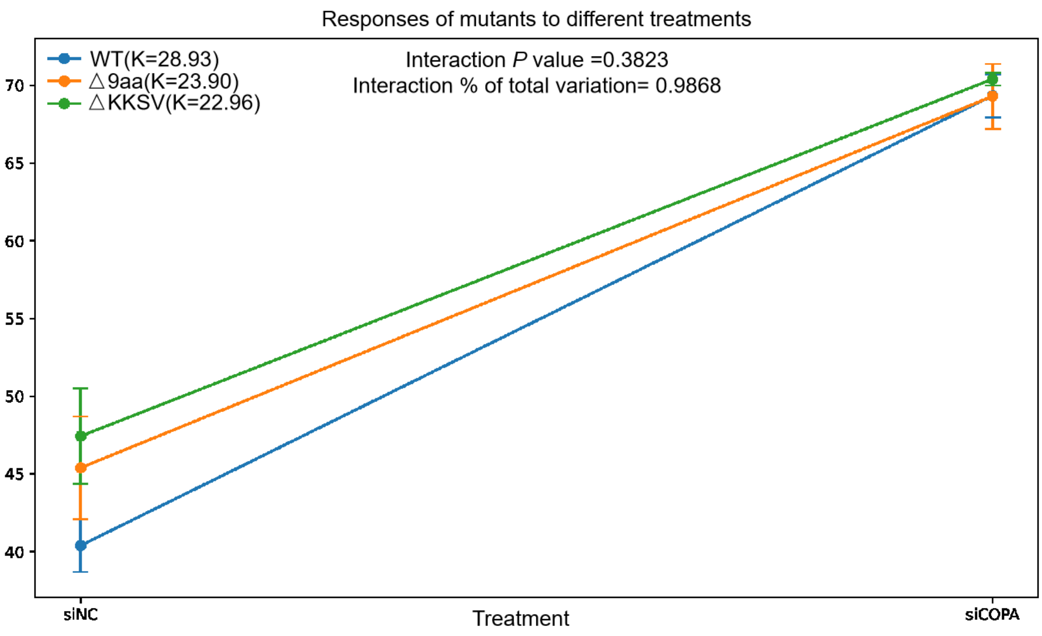

FIG S2 Responses of S protein and mutants to siCOPA.

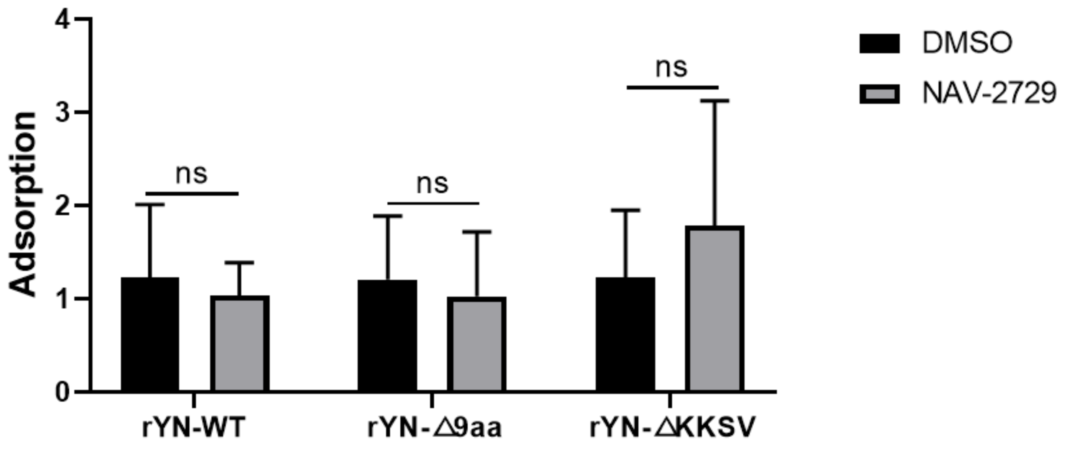

FIG S3 Evaluation of the adsorption capacity of IBV by NAV-2729. CEK cells were pre-treated with 10 μM NAV-2729 for 1 hour prior to infection with rIBVs; CEK cells were incubated with each viral strain at an MOI of 1 at 4 ℃ for 1 hour. After ten washes with cold PBS, the relative genome levels were quantified using RT-qPCR.

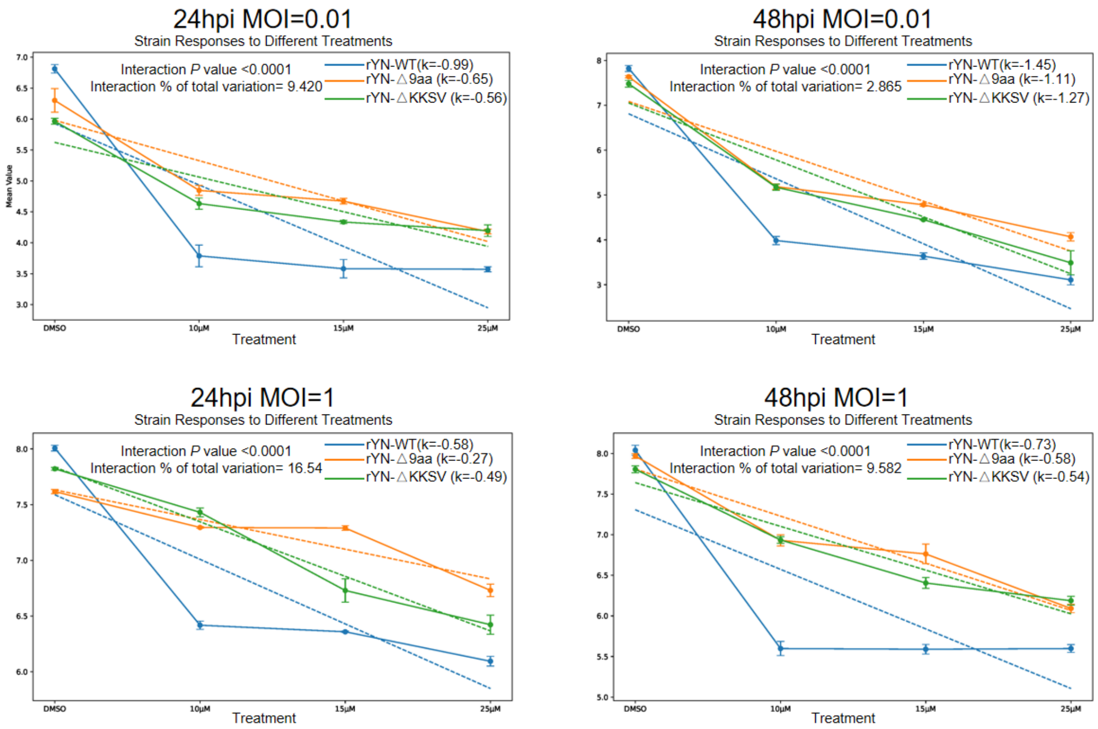

FIG S4 Responses of different rIBVs to different treatments.
